# Enabling the Voices of Marginalized Groups of People in Theoretical Business Ethics Research

**DOI:** 10.1007/s10551-021-04973-3

**Published:** 2021-10-16

**Authors:** Kristian Alm, David S. A. Guttormsen

**Affiliations:** 1 USN School of Business, Department of Business, Strategy and Political Sciences, University of South-Eastern Norway, Mailbox 7053, N-3007 Drammen, Norway; 2 Department of Communication and Culture, BI Norwegian Business School, Nydalsveien 37, N-0484 Oslo, Norway

**Keywords:** Marginalized people, Business ethics, Scientific knowledge, Epistemology, Normative

## Abstract

The paper addresses an understudied but highly relevant group of people within corporate organizations and society in general—the marginalized—as well as their narration, and criticism, of personal lived experiences of marginalization in business. They are conventionally perceived to lack traditional forms of power such as public influence, formal authority, education, money, and political positions; however, they still possess the resources to impact their situations, their circumstances, and the structures that determine their situations. Business ethics researchers seldom consider marginalized people’s voices and experiences as resources to understand their lives, as demonstrated through a review of 7500 articles published in the *Journal of Business Ethics* and *Business Ethics Quarterly* (2000–2019). Only 78 studies included aspects of marginalized groups. 69 of those studies discussed the topic of marginalized groups of people, but without integrating their explicit voices into the text. Only 9 of the 78 articles featured marginalized people’s explicit voices about their marginalization experiences incorporated into the text as a source for exploration. None of the identified studies discussed the potential for theorizing based on such voices. This paper contributes to business ethics theory by developing four theoretical possibilities vis-à-vis the critical voices of marginalized people’s experiences in business: (a) marginalized theory on critical agency and freedom of speech; (b) the gatekeeping role of academia; (c) primary sources; and (d) a participative perspective. Discussing the theoretical potential of quoting the above voices can enrich business ethics research in terms of the theoretical understanding of marginalized groups in business.

## Introduction

There is a shortcoming in business ethics research regarding the roles of marginalized groups of people (MGP) in business organizations and in particular their own narration of lived experiences concerning marginalization in business. MGP’s voices seldom appear in the scientific literature published in the field’s leading scholarly journals, herein the *Journal of Business Ethics* (*JBE*) and *Business Ethics Quarterly* (*BEQ*).

In scholarly research, MGP have been defined as people who lack the ability to use traditional forms of power—such as public influence, authority, education, money, and political positions—to affect their situations and the structures that determine said situations (Shepheard-Walwyn, 2018). Our definition is inspired by contributions on intersectionality, power, and democratic participation (Collins, 2017); status inconsistency (Meeks, 2003); freedom of speech for MGP (Bluden, 2004); and the dynamic force of MGP’s counter public spheres (Habermas, 1989), considered from a feminist perspective (McLaughlin, 1993).

In the current theoretical paper, we consequently define MGP as people who lack one or more types of such traditional power and being to some degree powerless in one or more contexts, but more or less powerful in others—nevertheless without repealing their marginality. Thus, we consider MGP as being able to produce different degrees of resistance and thereby to limit the lack of power and discrimination they might experience in relation to the aforementioned characteristics. Such resistance can vary based on different degrees of resilience and resources. Marginalization might occur due to various characteristics, such as discrimination based on race, age, gender, lifestyle, or economic status; lack of access to education; or living in a geographically isolated area or deprived community (Shepheard-Walwyn, 2018; United Nations Development Programme, 2017).

MGP have a large presence within business organizations and wider society. In combination with the hardships many MGP have faced and are currently facing, coupled with the diminutive focus on these groups within business ethics research, there is a significant need to investigate, and to produce scientific knowledge about, MGP as a key business phenomenon. There is an increasing international effort, echoed in the business world, to alleviate the disadvantages and discrimination faced by MGP (Bansal, 2002; Deloitte, 2020; Sustainable Development Goals, 2020; United Nations [UN], 2015). Following the global financial crisis (2007–2008), the growing gap between the world’s wealthiest and poorest people increased the number of MGP and worsened economic inequality for those already living at the margins. The ongoing COVID-19 pandemic has exacerbated these negative trends. More and more MGP must combat the effects of layoffs and job losses at an unprecedented rate, in addition to facing a looming global economic downturn (Vera, 2020).

However, MGP remain understudied within business organizations. In the business ethics field, research on MGP predominantly includes studies of people who have been marginalized due to a particular disadvantage related to their job situation (e.g., Gao et al., 2016; Kurland, 2001; Lucas et al., 2013; Snyder, 2010). In addition, only a handful of studies have featured insider perspectives through quotations of oral or written speech of MGP that provide important access to their understanding of their role in business (e.g., Kates, 2015; Klettner et al., 2016; Olabisi et al., 2019; Terjesen & Sealy, 2016). Although sophisticated scientific theories and methods dominate the MGP discourse, this discourse is limited to narration by authors with outsider perspectives.

The missing insider perspectives on MGP in business ethics research highlights several shortcomings. First, it leads to a lack of discoveries about MGP and the dynamics of their existence, despite their having challenges, needs, and possibilities for transcending their limits placed upon them. Second, it deprives the business ethics field of theoretical opportunities to improve scientific knowledge production about MGP’s view and criticism of their experience of being marginalized in business—not only in terms of their roles in business but also in the understanding of the accompanying challenges, resistance, and progress when it comes to being powerful as an opposition to this view—as well as the prospect of theorizing on the consequences of failing to explore such possibilities. Voices as quotations that appear in the text are completely absent from the dominant discourse, and consequently, the dominant discourse treats MGP’s voices as theoretically worthless. Third, the dearth of insider perspectives, narrated by the MGP themselves, concerning marginalization in business leads to missed opportunities to theorize about this knowledge as expressed in their own voices. By “their voices”, we mean MGP’s explicit viewpoints and criticism of their experience of being marginalized in business. Fourth, the assumptions underlying the dominant discourse are misleading. The insider perspective of MGP’s voices on their experience of being marginalized in business represents high scientific value because it creates possibilities for developing theoretical concepts that could enrich and renew the application of dominant theories and methods. Fifth, due to this lack of focus, the needs and insider perspectives of MGP seldom reach their managers.

The purpose of this article is to theoretically examine, and to theorize about, the missed opportunities for scientific knowledge production concerning MGP’s voices in terms of personal lived experiences of marginalization in business, due to such voices being overlooked in the *JBE* and *BEQ* during the 2000–2019 period. The overlooked voices and viewpoints of MGP are expressed through various means and cultural representations—most directly through speech acts as quotations, and to a lesser degree via artifacts, rituals, actions, and written documents.

Our objective is to theoretically examine the knowledge potential of these voices, providing important access to MGP’s understanding of their marginalized role in business. We concentrate on speech acts as quotations, but we are not of the opinion that voices can only be expressed in direct quotes, as highlighted above. However, in this paper, we have limited our exploration of the epistemological value of MGP’s voices concerning personal lived experiences of marginalization in business to direct quotes based on reasons expressed in the theory on counter public spheres with the capacity to challenge domination (Downey & Fenton, 2003; Habermas, 1992); marginalized theory on critical agency (Blunden, 2004; Sen & Drèze, 2002); decolonizing perspectives on the righteousness of uplifting standpoints considered unworthy (Go, 2017; Morris, 2017); and perspectives on a more inclusive function of the gatekeeping role of academia (Crane, 1967; Hojat et al., 2003).

Thus, we pose the following research question:

When considering MGP’s voices expressed in quotes relating to the understanding of their roles and lived experiences of being marginalized in contemporary business, what knowledge production possibilities arise, and what types of concepts ought to be applied?

To achieve the above, our paper features a somewhat unconventional structure, as it does not take the form of a “pure” literature review, empirical study, or theoretical paper. We provide, first, a scoping literature review that highlights the main approaches to researching MGP in the leading business ethics scholarly journals. This section explicates the method employed for conducting the review and subsequently identifies the diminutive focus on MGP topics and explicit voices in extant business ethics research. This serves as a foundation to point out areas of missed opportunities to theorize about MGP’s explicit voices. Second, we provide a background of the MGP phenomenon that we investigate—with a particular focus on why the outsider perspective appears to be the dominant discourse in the business ethics field. Third, in the main section, we elaborate on four theoretical opportunities to increase the research focus on MGP’s voices and theorize about the scientific knowledge that researchers can produce regarding MGP. We conclude by highlighting our theoretical contributions, the study’s limitations, and avenues for future research.

## Literature Review: Identifying a Diminutive Focus on MGP

Below, we will first explain the design of the conducted literature review by outlining the scope of the review, the rationale for the time frame, the method, and the analysis approach. Second, we will identify the main trends in extant business ethics research concerning MGP.

We conducted a carefully designed literature review to serve the purpose of the current paper, which can be labeled a scoping review. This type of review belongs to the broader group of systematic reviews due to focusing on obtaining an overview of a research field (through mapping), as well as associated gaps in extant literature, but in a systemized manner that is also transparent and replicable (Arksey & O’Malley, 2005).

### Scope

Because we chose to study only two journals in this field, we acknowledge that we might have excluded relevant work on MGP from our analysis. However, we argue that our focus on these two leading journals is well founded due to these journals plausibly exercising the most influence and reflecting the breadth and depth of the research field. The *JBE* and *BEQ* are the two highest ranked business ethics journals in the Chartered Association of Business Schools’ Academic Journal Guide and on the Australian Business Deans Council’s Journal Quality List, and the *JBE* is on the list of Financial Time’s top 50 Journals.

We surveyed the entire population of articles published by the two journals (approximately 7500 articles) during the chosen period. The aim was to identify published articles that related to MGP and to obtain an understanding of how MGP have been studied in leading business ethics journals. MGP were understudied in leading business ethics journals (i.e., the *JBE* and *BEQ*) during the 2000–2019 period.

The rationale for choosing speech acts is twofold. First, in our sampled articles, quotations were evidently the avenue by which MGP most freely expressed their narration of their own lived experiences of marginalization in business. Second, as later explained, our analysis of sampled articles did not generate any outputs in which the MGP’s voices featured their own narration concerning lived experiences of this marginalization. On the contrary, other persons’ (scientists’) viewpoints about the MGP constituted the modus of expression; thus, MGP were limited to expressing themselves only indirectly, leading to a potentially biased representation. Consequently, the reason for focusing on personally experienced marginalization is due to the risk of losing opportunities for theorizing based on self-narration of actual lived experiences.

### Rationale for Time Frame

The time frame of our review was demarcated to include no more than the two most recent decades, as our argument about the importance of investigating MGP is partly contingent on the recent/current societal context. The last two decades reflect a period during which MGP increasingly became part of work organizations’ agendas (see Morris, 2017). For example, the focus in business and management research regarding MGP was, indeed, intensified during the aforementioned period, contemplating the UN implementing, in 2015, the Sustainable Development Goals, which, in essence, focus on reducing global inequalities. Furthermore, research interest has increased on marginalized topics linked to the wider dialogs in society about sexual orientation, gender, race, religion, and ethnic origin—partly related to understanding and stopping hate speech (Relia et al., 2019). The decision is also supported by our initial searches in both journals, where publications on MGP did not appear until the early 2000s.

### Method: Identifying Relevant Articles

The first stage of the review process involved identification of the relevant articles to include in our sample. To be eligible for inclusion, an article had to fulfill the following criteria: (i) published in the *JBE* or *BEQ*; (ii) published between 2000 and 2019; and (iii) addressing a topic relating to MGP and/or an aspect of marginalization. Inclusion for the latter criterion reflected the applied MGP definition that we presented in our Introduction (i.e., people who lack the ability to use traditional forms of power—such as public influence, authority, education, money, and political positions—to fundamentally affect their situations and the structures that determine said situations, but with different degrees of resilience) (Shepheard-Walwyn, 2018).

To identify relevant journal articles, we made use of relevant databases (e.g., Web of Science), searching with various strings of keywords (e.g., “marginal” and “minority,” including the use of different search functions and prefixes). We prepared an Excel spreadsheet for a research assistant to utilize in this process (recording included articles).

However, this approach was deemed insufficient to identify the particular types of articles we wanted to investigate. Being aware that MGP might be referred to using different terms, and that some articles might address marginalization as a phenomenon but not necessarily label it as such, we opted for browsing through every page in each of the journals’ issues, amounting to several hundreds of thousands of pages of text. This accurate approach allowed for applying a qualitative assessment to decide if an article related to MGP or not; if so, the article would be included in the sample. The results of browsing each page in each issue were reported on the spreadsheet, and we conducted several checks of the research assistant’s work to ensure consistent and appropriate sampling.

Additionally, we utilized the databases to determine whether the manual approach had left out any relevant articles. This was not the case; conversely, with the chosen approach, we were able to identify many relevant articles not picked up by database searches. This stage of the review generated 78 articles that met the inclusion criteria.

### Mapping and Analysis

The second stage of our review process encompassed categorization of the abovementioned 78 articles. We added a key inclusion criterion related to what evidence from the articles we considered to constitute, and reflect, the mentioned MGP’s voices. We decided to focus on speech acts (that is, quotes); nevertheless, we recognize that opinions and narration about MGP can also be expressed through other means and social phenomena, such as ritual traditions, intended actions, and written documents.

We employed an exploratory frequency analysis (O’Leary & Sandberg, 2017) in accordance with the principles of a deductive research paradigm. We predetermined the number of categories with which the sampled articles were to be associated and subsequently counted. We found it appropriate and pertinent to establish two categories, replicated for both journals (i.e., articles within which the voices of MGP are included [through quotes], and articles in which their voices are not included but they are generally referred to as a group, with MGP being part of the paper’s theme).

The above was mapped using the spreadsheet, on which we recorded additional variables from an article’s content, such as the geographical location of the empirical foundation, key topic(s), methodology, and theoretical framework. However, only nine articles included MGP voices and understandings of their role in business, as well as their own narration and criticism of personally experienced marginalization. This narration, we found, only occurred through quotations. These researchers publish about MGP, even if they make use of direct quotes from an ‘insider perspective’, simply because the quotes are about MGP. The remaining 69 articles (out of the 78 initially recorded) discussed important problems at the workplace of MGP. Even if the authors did not use quotes of MGP to discuss this topic they apparently published about MGP when including the voices of MGP. The 69 articles were also reviewed with the objective of exploring whether the MGP’s lived experiences regarding marginalization were conveyed through means other than using quotes (e.g., by analyzing an artifact, a written diary). Our review did not identify such means; hence, in the current paper, we argue that quotations, as speech, are a relevant unit of analysis.

We also utilized exploratory frequency analysis to count the articles in relation to different time periods and which of the two journals published them. 78 is a marginal proportion of 7500—even though the number increased throughout the surveyed period, especially during the last 10 years (Table 1).

**Table 1:** Frequency of publication of studies on marginalized people in the JBE and BEQ

Time period	JBE	BEQ	Total (per year)
2015–2019	38	5	***43***
2010–2014	16	3	***19***
2005–2009	8	0	***8***
2000–2004	6	2	***8***
Total (per journal)	***68***	***10***	

### Main Trends: MGP and Business Ethics Literature

According to the frequency analysis, the most discussed topic in the 78 articles related to workplace bullying (e.g., Bulutlar & Öz, 2009; LaVan & Martin, 2008; McAllister & Perrewé, 2018; Samnani & Singh, 2016; Valentine et al., 2018). The other most discussed problems included abusive supervision (e.g., Avey et al., 2015; Henle & Gross, 2014; Zhang & Bednall, 2016) and women confronted with challenges from boards (Burgess & Tharenou, 2002; Carrasco et al., 2015; Du, 2016; Terjesen & Sealy, 2016). The MGP thematized in these studies were located primarily in Western societies.

Topics discussed with much lower frequency encompassed Islamic female entrepreneurs (Tlaiss, 2015), sex discrimination (Lin & Ma, 2016), microfinance (Beisland et al., 2019), the silencing of female workers in Tanzania’s mining industry (Lauwo, 2018), garbage workers in England (Hamilton et al., 2019), workplace sexual harassment in China (Xin et al., 2018), migrant workers in Australia and China (Lucas et al., 2013; Underhill et al., 2018a, b), and poverty alleviation (Van Sandt & Sud, 2012). The MGP thematized in the above studies were located in Western and non-Western countries.

The interest in MGP in the *JBE* and *BEQ* increased during the selected period (Table 1). This might be attributed to the influence of trends such as responsible research and responsible management agendas, the UN’s Sustainable Development Goals (launched in 2015), and a concern shared by many in society since the financial crisis that economic and political marginalization might contribute to political polarization and extremism. International commentators are now focusing on the risk that the COVID-19 pandemic has contributed to the growing gap between Wall Street and the real economy, as well as between rich and poor people worldwide, which might lead to an even stronger interest in MGP in the two journals in the near future (The Economist, 2020).

The 78 articles can be divided into two types of discourses: first, the dominant discourse, indicating that MGP’s voices have not been included (69 articles), and second, the nondominant discourse, where said voices have been incorporated into the text (9 articles).

The authors of the nine articles incorporated MGP’s voices of their own lived experiences of marginalization in a business context as a significant component of the scientific knowledge production (Table 2), and their understanding of these voices aligns with our definition (see paraphrasing below). The *JBE* is the main arena for this discussion. The only article of this type published in *BEQ* examines various ways “through which caste, dirty work, and dignity intersected in the narrative accounts of Dalit janitors” (formerly known as “untouchables”) (Mahalingham, 2019, p. 213).

**Table 2:** Studies published in the JBE and BEQ in which the voices of marginalized people are heard

Time period	JBE		BEQ	
	Voice as important access (quote)	Voice as indirect access (thematic)	Voice as important access (quote)	Voice as indirect access (thematic)
2015–2019	6	32	1	4
2010–2014	1	15	0	3
2005–2009	0	8	0	0
2000–2004	1	5	0	2
Total (per journal)	***8***	***60***	***1***	***9***

One of these studies in the *JBE* interpreted the voice of the leader of an East African indigenous group, describing how his people emancipated themselves from a non-stakeholder position threatened by extinction and became primary stakeholders by cooperating with a Spanish multinational corporation on a successful project (Olabisi et al., 2019). Another article focused on the viewpoints of hotel workers with intellectual disabilities regarding their employment experiences of freedom and anxiety, operating in a supportive working environment (Meacham et al., 2019). Another group of authors examined how garbage workers respond to ascriptions of servility through discourses of everyday heroism (Hamilton et al., 2019). One article discussed women’s viewpoints on disproportionate changes between the higher number of women on boards and the consistently low number of women in senior executive positions in Australia (Klettner et al., 2016).

Furthermore, two authors gave voice to underrepresented stakeholders, arguing that, in times of crisis, a corporation’s position of power should not marginalize or altogether silence alternative discourses (Dunn & Eble, 2015). Another study interpreted the voices of Muslim women entrepreneurs, describing how they understood and conducted their business in the Arab world based on Islamic business ethics (Tlaiss, 2015). In addition, one article examined the existential perspectives of migrant workers regarding how the structure of Foxconn Company imposed unique indignities on them in a Chinese factory (Lucas et al., 2013). Finally, one study analyzed the viewpoints of female nurses in the United Kingdom regarding negative gender-role stereotypes that pervaded their careers (Lane & Piercy, 2003).

Importantly, regarding our contribution in the current article, we found no studies among the identified 69 that discussed or theorized on the prospect of creating theoretical possibilities for scientific knowledge production. Neither did we find this in the nine articles that feature MGP’s voices; the authors instead considered these voices mainly as an instrument for conveying a more limited type of knowledge (about the particular working life contexts recently mentioned) but not as an avenue for producing comprehensive theoretical knowledge on universal or general phenomena.

### The Negative Power of a Scientific Paradigm

In this section, we focus on why the explicit voices of MGP were not included in approximately 7500 articles in the *JBE* and *BEQ* over two decades—in relation to the dominating paradigm of objectifying in science that due to researchers’ paradigmatic positioning has led to excluding MGP’s explicit voices. We identify seven plausible reasons to legitimate this exclusion. We interpret such exclusion as a comprehensive use of negative power with the ability to stop important activity (Rus, 1980). We do this to challenge a scientific hegemony and to argue for a paradigmatic shift. The following theoretical discussion is an attempt to pursue such a shift.

First, along with traditions of positivism, the authors of the 69 articles, which admittedly focus on MGP topics but fail to incorporate their explicit voice, might have assumed that avoiding the bias of the insider perspective and achieving an alleged objectivity of the outsider perspective contribute to a correct interpretation. Second, the dominance of quantitative methodology might have played a role. 35 of the 69 studies employed quantitative methodology involving “extracting the research subject from its concrete context and ‘decomposing’ it into variables” (Piekkari et al., 2009). Such “decomposing” could be reassuring of the nonreflexive material of their words, which Merleau-Ponty called the body “as an alternative, non-cognitive and pre-reflexive source of individual ‘knowing’” (Mahadevan, 2015). Second, MGP’s explicit voices are considered neither commercially valuable nor important resources for the main strategy of the firms in the 69 articles. This might be an indication of a reason their voices do not qualify much for scientific interpretation (Lahroodi, 2007; Rawwas et al., 2013).

Third, a comprehensive interest in both journals throughout the two decades is the interdisciplinary discussion of the commercial, strategic, ethical, and political significance of the management strata of the business organization, and a corresponding lack of discussion about the significance of the lowest strata of the business corporation. Fourth, patriarchy might have played a role as an exclusionary type of discourse (Poupart, 1997). This implies that the explicit viewpoints coming from the lower strata of the business corporation are associated with considerably less significance compared with viewpoints emanating from the upper strata, because the power of the former is more limited.

Fifth, MGP might have had limited access to scientific careers and opportunities, making them less visible or recognized in the mainstream of science (Sumerau, 2016). Sixth, the exclusionary gatekeeping role of editors, reviewers, and authors has most probably, however unintentionally, played a role (Crane, 1967). Seventh, studies on researcher positionality concerning self-referential aspects of research could indicate an important reason (Corlett & Mavin, 2018). When a researcher belongs to a marginalized group, there is a possibility to use an autobiographical perspective or other methodologies explicating their researcher positionality. However, we did not find any examples of these two types of researcher positionality. This lack of both autobiographical and other methodologies that could elucidate the researcher’s marginalized position might be a sign of self-marginalization, and as such it might contribute to MGP voices and topics being marginalized in the *JBE* and *BEQ*. This assumption is, however, based on the reasonable presupposition that MGP are among the authors publishing in the *JBE* and *BEQ* (2000–2019).

However, we acknowledge that quoting their voices does not provide direct access to MGP perspectives. Our quoting is unavoidably linked to theoretical, methodological, and often selective uses of their voices, different from the original use, function, and intention of the voices, a difference that might hinder the correct or authentic understanding of these originalities. We should employ these scientific instruments to develop a self-correctable preunderstanding of the original context, being sensitive to the original use, function, and intention, to develop a better, and hopefully important, but still correctable, understanding of the quoted voices of MGP.

## Discussion of Theoretical Possibilities: Normative Epistemology

In this main section, we examine the theoretical knowledge potential of the insider perspective of MGP’s voices regarding their own lived experiences of marginalization in a business context, which was overlooked in the *JBE* and *BEQ* during the 2000–2019 period, as demonstrated in our literature review. Anchored in normative epistemology, we seek to highlight the missed opportunities for theorizing on MGP’s voices on their lived experiences of being marginalized in business. Thus, we explore four ways of theorizing about MGP’s voices as a criticism of being marginalized in business, a criticism most clearly expressed in quotations: (i) We apply Amartya Sen’s perspective on MGP’s critical agency to contribute to a more positive publishing policy in the *JBE* and *BEQ*, in cooperation with MGP, a plan focusing on MGP’s explicit voices about their experiences of being marginalized in business; (ii) we present a criticism of the gatekeeping role of academia linked to a possible exclusion of the quotations of MGP’s critical agency; (iii) we denote quotations of MGP’s criticism of their marginalized role in business assigned the role of a primary source; and (iv) we introduce a participative perspective on MGP’s criticism of the lived experience, particularity, and complexity of being marginalized in business. We consider these heterodox positions as characterized by what Dobusch and Kapeller (2012) call “the epistemological advantage associated with a pluralist conception of science” (p. 485).

Because the phenomena analyzed in social sciences are subject to the contingency of history and culture, in accordance with this study, it is probable that it would be an advantage to use a variety of analytical approaches and concepts to interpret more rigorously and produce more robust knowledge about the chosen social phenomenon of MGP’s lives. They are parts of a reality we as social scientists have limited possibilities to understand. Thus, within this complexity, MGP’s own understanding of their lives and experiences should play a key role, in order for the social scientist to gain a robust and relevant preunderstanding, however preliminary and thus disputable. This heterodoxy of our positions, as to epistemology, is chosen exactly because they converge toward an analog or familiarity of normativity—opening possibilities for MGP to enhance their critical voices of their marginalization in business and enhance positive alternatives by letting their voice on this topic be better heard.

With this pluralist approach, we also aim to avoid continuing to treat MGP as voiceless objects when studying their roles in contemporary business. Instead, we consider their voices a principium for greater critical agency and freedom of speech for MGP (Blunden, 2004; Sen & Drèze, 2002), i.e., as a point of departure for further reflections about the topic in question, but based on the possibility of a more positive publishing policy in the *JBE* and *BEQ*.

We distinguish between normative and descriptive or naturalistic epistemology (Kitchener, 1994). The descriptive epistemology tradition explores what researchers do, whereas the normative epistemology tradition is centered on what *should* be the concepts, explanations, theories, and methods of scientific exploration, and what requirements researchers *should* fulfill (Grimen, 2004). Our interest is in normative epistemology, primarily due to a lack of research on MGP voices that should be corrected. Additionally, we did not find any epistemological studies of MGP in business ethics, only epistemology on managers (e.g., Lahroodi, 2007; Rawwas et al., 2013). The potential of MGP’s voices for theoretical knowledge production raises questions regarding which scientific concepts can take their voices seriously as a point of departure for scientific exploration.

We argue that theorizing about, on the one hand, the institutional preconditions for MGP’s voices (most clearly expressed in quotations) linked to Sen’s theory on critical agency and to the gatekeeping role of academia, and on the other, the positive evaluation of these types of MGP’s critical agency understood as primary sources and described in a participative perspective fulfill this need.

However, to present a more profound exposition, in the cross-disciplinary landscape of normative epistemology, we delimit the inquiry to a normative epistemology, but not including the phenomenology of “the meanings things have in our experience” (Stanford Encyclopedia of Philosophy, 2013). A normative epistemology capable of developing scientific concepts based on the potential of MGP voices could be established in several theoretical ways; we mention a few influential ones.

Justification could be linked to a proponent of modern critical theory, Habermas. The legitimizing force of the scientific concepts and the corresponding scientific value of MGP’s free voices and topics would be to challenge scientific journals as part of a dominant media culture to open the journals to MGP’s voices and topics as part of counter public spheres. In addition, MGP used this possibility and thus experienced greater freedom of speech—the core value of democracy—regarding their own lived experiences of marginalization in business, with the “capacity for challenging domination” (Downey & Fenton, 2003, p. 183).

Habermas discussed the forces of free speech of counter public spheres, such as those occupied by movement-based or activist groups. He posited that the mass media public sphere “may be subject to periodic crises that may be exploited by groups in civil society” (Downey & Fenton, 2003: 189) to make their voices heard. Calhoun (1992) criticized Habermas’s Adornian-inspired pessimistic position from the early 1960s, *The Structural Transformation of the Public Sphere*. Calhoun assumed that the function of mass media was not massively negative. There is certain room for alternative democratic strategies. Groups in civil society can influence the mass media and establish “alternative, discursively-connected public spheres” (Calhoun, 1992, p. 37). Interestingly, Habermas revised his public sphere thesis and took account of such possibilities (1992, p. 427).

Consequently, in line with Habermas’ revised position, we argue that the free voices and topics of MGP, as belonging to civil society, have epistemological value as criticism coming from counter public spheres challenging the dominant culture of scientific journals, to make their voice and MGP topics better heard.

One important way to make their voices better heard as participants coming from counter public spheres could be to articulate well-founded critical arguments on how to produce more just social arrangements in the dominant public sphere of scientific journals. Such critical voices might challenge the hegemonic and elitist opinions of the editors, reviewers, authors, and readers, which primarily are based on outsider perspectives.

The justification could also be that considering the voices of MGP would serve as a criticism of the reproduction of knowledge hierarchies, turning them “upside down,” based on a decolonizing position (Morris, 2017). Go (2017) addressed the challenge of reproducing social inequality in epistemology and argued that we should consider “the question of knowledge hierarchies; of how certain standpoints get marginalized as inferior, unworthy, and lesser while other standpoints get valorized as superior” (p. 194). Like Go, we criticize that business ethics studies reproduce such knowledge hierarchies of our societies when they consider (however unintentionally, but probably as something to be expected) that “our” MGP voices are epistemologically unworthy. We also underscore that MGP could benefit from the scientific majority listening to and giving prominent place for MGP’s perspectives on new types of social arrangements that have the capacity to take their voices more seriously. They would then have the advantage of challenging traditional knowledge hierarchies and their social structures, and their voices could then be considered to be of high scientific value, hopefully contributing to social change.

### Marginalized Theories: Freedom of Speech and Critical Agency

Marginalized theories formulated by researchers with marginalized backgrounds are relevant to our normative epistemology. This academic field is comprehensive and complex, including theories such as feminism, race, and decolonizing theories. Recently, the academic and theoretical consciousness concerning the existence of marginalization and marginal voices as part of academic institutions has been growing (Morris, 2017).

Amartya Sen’s theoretical and empirical contributions on potential freedom, economy, and the burdens of MGP are among the influential contributions of marginalized theories. Sen’s biography is closely linked to this academic effort. When he was 14 years old, he witnessed India’s last famine in 1947, during which two to three million people died. Ever since growing up in the former British colony, the liberated and free nation of India, Sen has been intellectually occupied with the life struggle of MGP—through his own lived experiences—and the economic, political, and ideological presuppositions they ought to have to live dignified lives.

Sen’s theoretical contributions on critical agency are relevant for understanding the knowledge potential of the voices of MGP in question. Several scholars underscore the significance of critical agency in Sen’s thinking. Poveda and Roberts (2018) underscore Sen’s argument that critical agency to question and reject unjust social norms is pivotal in tackling inequalities of any kind. As an overview of the main trends in Sen’s theories, Blunden (2004) concludes in the same way with the following:

The whole point is that to the extent that people have a critical voice in the social arrangements determining their own life, then they can determine those arrangements in collaboration with others affected by those same social arrangements. (p. 15)

Blunden refers to social arrangements such as poverty and the institution of “son preference.” Sen underscores that son preference in India and China has led to the abortion of female fetuses, to the degree that 40 million people are “missing.” He showed that this problem increased with industrialization and rising real incomes. The increase took place even in cultures where “women had a voice.” Even women who are educated and those who have full control over “the decision whether or not to abort a female fetus may be active participants in exercising son-preference because they share their husband’s preference for a son” (Blunden, 2004, p. 5). According to Sen and Drèze (2002),

Strengthening women’s agency will not, by itself, solve the problem of ‘son preference’ when that works through the desires of the mothers themselves. (p. 258)

(…) it is possible to overcome the barriers of inequality imposed by tradition through greater freedom to question, doubt, and—if convinced—reject. An adequate realisation of women’s agency relates not only to the freedom to act but also to the freedom to question and reassess. Critical agency is a great ally of development. (p. 274)

In our perspective, it is of key significance that Sen advocates for critical agency as greater freedom to speak and to act for MGP as important factors to oppose their experience of being marginalized. The marginalizing effects of social inequalities such as son preference, poverty, and racism could better be solved for the persons involved based on such a critical agency of MGP, as a point of departure—their questioning, doubting, reassessing, and rejecting. However, in China, where the country’s one-child policy has played an important role in the “son preference,” it will arguably be a long-term project to oppose and overcome a tradition through freedom of speech that indirectly is guaranteed by law, if possible.

Additionally, as Walker (2005), Pressmann and Summerfield (2000), and Marginson (2011) underscore, Sen argues that to enhance such critical agency, an individual must have certain capabilities to take advantage of the supportive social conditions, such as the values and possibilities of education, together with economic and social resources, such as political and civil rights. Marginson (2011) underscores that greater critical agency requires better social conditions that permit and support the exercise of opposing the marginalized experience, as a sign of a “deep complementarity.” He also underscores this progress as necessary if “persons formerly excluded are to gain access and sustain effective presence within higher education” (p. 30).

According to Marginson (2011), this should take place through interventions in institutional processes where persons formerly excluded or underrepresented are empowered and resourced, becoming their own best advocates. We consider the relevance of Sen’s theory on improving the critical agency of marginalized groups (for an understanding of the knowledge potential of the voices of MGP in question), with the theory’s focus being on social systems such as higher education improving the support of MGP’s critical agency and their critical agency taking advantage of this improvement, thus influencing such systems.

We thus recommend, as an expression of our normative epistemological perspective, that the *JBE* and *BEQ*—as powerful institutional representatives in the discourse of higher education—broaden the discussion and enhance their knowledge production. This should take place in cooperation with MGP, which effectively would pave the way for a paradigmatic shift enabling an increase in studies about MGP’s explicit voices on their experiences of marginalization in business. This could support their critical agency to expand their freedom to act and freedom of speech to better oppose the marginalization they experience in business. The *JBE* and *BEQ* should introduce what Walker (2005) calls a strategy of educational action research characterized by the involvement of “all those affected … engaging in action and deliberative reflection together” (p. 109). The main editors of the *JBE* and *BEQ*, relevant section editors, reviewers, and relevant authors could invite MGP from different counter public spheres, or they could invite themselves, to discuss a more positive editorial policy enhancing articles that express the critical voices of MGP concerning their experiences of marginalization in business. As part of this action research program, MGP could, as a first step, be invited as authors, coauthors, reviewers, and guest editors of a special issue. MGP could promote the explicit voices of other MGP expressing their critical agency on being marginalized in business (their own words, quotations). This cooperation or complementarity (Marginson, 2011) could be a sign of MGP being the best advocates of challenging the hegemonic paradigm that refrains their voices from being heard. This cooperation would also imply that the empowerment of persons formerly excluded or underrepresented would give them the possibility for exactly that: to act as their own best advocates (Marginson, 2011).

Our recommendation of such a new, more positive publishing policy where MGP promote the explicit critical voices of MGP aligns with our first normative epistemological perspective, therein our earlier discussion of Habermas’s revision of his public sphere thesis (1992, p. 427). The *JBE* and *BEQ* could, *as* dominant scientific journals, open the door for voices from counter public spheres. MGP’s critical voices often arise from just such more peripheral social arrangements compared with the more dominant powers of society. This would hopefully create better institutional preconditions for MGP and their counter public spheres, thus experiencing both greater freedom of speech and action and better possibilities to critically consider personal lived experiences of marginalization in business, with the “capacity for challenging domination” (Downey & Fenton, 2003, p. 183).

This would, however, be a modification of our earlier focus, in our normative epistemological perspective, on groups in civil society exerting influence on the mass media and establishing “alternative, discursively-connected public spheres.” The influence this time would occur inside and in explicit cooperation with two representatives of dominant media, not outside, as Habermas presupposes, and thus not upon traditional mass media, but inside leading scientific media as a paradigmatic shift.

Our recommendation of an enhanced positive publishing policy also aligns with our second normative epistemological perspective (Go, 2017; Morris, 2017), but in a modified way. MGP enabling the critical voices of MGP might then serve as a decolonizing criticism of the journals’ reproduction of the knowledge hierarchies we identified in our literature review—not turning them “upside down” (Morris, 2017), but at least contributing to a more just MGP policy. This new and more just policy would thus change the journals’ reproduction of social inequality in epistemology by addressing “the question of knowledge hierarchies; of how certain standpoints get marginalized as inferior, unworthy, and lesser while other standpoints get valorized as superior” (Go, 2017, p. 194).

### The Gatekeeping Role of Academia

A more positive publishing policy could be explicitly achieved based on MGP questioning, doubting, discussing, and rejecting the gatekeeping role of academia (see Sen & Drèze, 2002). We assume that the exclusion of the quotations of MGP voices and topics in the *JBE* and *BEQ* transpired unintentionally. However, because academia often replicates the exclusion of MGP in the surrounding external society (Morris, 2017), this might imply an expected corresponding risk for business ethics journals when it comes to quotations of critical agency, topics, and voices of MGP. Discussion topics could be on the plan’s agenda.

The gatekeeping role of scientific editors and peer reviewers has been a longstanding discussion within academia (Hojat et al., 2003). Crane (1967) empirically demonstrates that editors of influential scientific journals serve as gatekeepers with respect to the evaluation of articles and tend to support the current orthodox views in their fields. She argues that their receptivity to new ideas and topics varies (Crane, 1967). Authors may, as we have shown, have had the same gatekeeping role as editors and peer reviewers when it comes to the deficiency of studies about the voices of MGP in business ethics research. Together, they probably produce a silent hegemony silencing (Crane, 1967).

However, editors, authors, and peer reviewers practice self-correction, constantly trying to improve the status quo, searching for new knowledge to correct the old. Nevertheless, the self-correcting system has come under serious scrutiny in recent years (Ioannidis, 2012). Thus, until business ethics journals and studies show a stronger willingness to integrate MGP’s free voices as quotations (e.g., through a new, more positive publishing policy where the *JBE* and *BEQ* cooperate with MGP) and discuss different aspect of their gatekeeping role (e.g., in a special issue), this self-correcting mechanism will not reach its true potential.

This more positive publishing policy, understood both as a public media platform enabling the critical agency of MGP and simultaneously changing the gatekeeping role of academia, would, according to our definition, have a double function. It would negatively serve to oppose MGP lacking traditional forms of power—such as public influence, authority, and participating in the discourses of higher education—to affect their situations and the structures that determine said situations (Shepheard-Walwyn, 2018). It would positively support MGP being able to produce different degrees of resistance and thereby to limit the lack of power and discrimination they might experience in relation to the aforementioned characteristics, by participating more significantly in the production of the significant self-correcting discourses of higher education.

One additional way for MGP to promote such a paradigmatic shift featuring a more positive editorial policy could be to use the special issue programmatically to denote the voices and viewpoints of MGP with the scientifically elevated role performing as a primary source. MGP would then give MGP the freedom to express themselves through their own narration. We present examples of how to do this in the next section.

### Primary Sources

According to Wadel (1990), the interpretations and explanations of ordinary social actors are often different from the interpretations and explanations of scientists. For example, the nine studies that included MGP voices as quotations are, in this fundamental aspect, different from the 69 studies of MGP that represent the dominant theoretical and methodological tradition. These studies did not vocalize MGP’s free voice and critical agency regarding their own lived experiences of marginalization in business in the text. Consequently, our literature review shows that quotations by MGP on this topic are low ranked as a primary source in *JBE* and *BEQ* between 2000 and 2019.

Consequently, scientists must consider how they should understand and denote the scientific value of social actors’ critical voices to evaluate such voices properly. In this regard, we consider a primary source as a medium of freedom of expression that explicitly expresses the voices and viewpoints of social actors, which means that they explicate the insider perspective. The reason for why the many different things mentioned as parts of the three following categories should be used as primary sources is thus that they explicitly express the insider perspective.

As part of a new, more positive publishing policy, MGP should programmatically promote the insider perspective by denoting MGP’s explicit voices/quotations as expressing the scientifically significant role of a primary source. This would be a way to operationalize a normative epistemology and make it productive. Primary sources, having the ability to express the insider perspective, occur in many forms. They are original testimonies and depictions in oral or silent forms as eyewitness narratives and accounts, but also interviews, conversations, fieldwork, internet communication, or firsthand observations both passive and participative, implying silent knowledge (Hox & Boeije, 2005; Ithaca College Library, 2016). Primary sources include also original testimonies and depictions in written form such as documents or artifacts created by a witness to or participant in an event, or an interview serving as a firsthand testimony or evidence (University of Washington Library, 2021), native texts, court proceedings, structured and unstructured diaries, as well as web and mail surveys (Hox & Boeije, 2005). In the internet age, primary sources appear as original testimonies in digital form such as administrative records; databases, internet archives, and existing digital records; in addition to images, sounds, and news archives (Hox & Boeije, 2005).

A normative epistemology quoting such a complexity of original and firsthand testimonies of oral, textual, and digital types (the list is not exhaustive), and thereby respecting their value, are providing authors and readers easy access to the significant information provided in MGP’s explicit voices about their criticism of their marginalized role in business.

Such a normative epistemology would then give rise to a wide range of secondary sources, meaning different types of scientific, cultural, and other interpretations of the primary sources mentioned, and thereby contribute to the public discussion of the legitimacy of MGP’s criticism and thus to pave the way for its legitimate breakthrough.

Access to such significant information from the primary source of MGP, through quotations, would provide marginalized authors (and others) with the possibility of attaining scientific independence, developing “their own knowledge, skills, and predispositions” (Singleton & Giese, 1999, p. 148). This significant information and independent interpretative approach could be about the critical agency of MGP, how MGP, through the quotations, “question, doubt, and‒if convinced‒reject” their role as marginalized in business (see Sen & Drèze, 2002, p. 274). Programmatically denoting this topic as worthy of the scientific significant reference; of primary source, could be one way of strengthening the publishing plan.

However, the use of quotations as primary sources for the advancement of greater critical agency and freedom of speech signifies no direct but important access to the original use and intention of their voices and experiences of critical agency. Consequently, researchers should cautiously interpret quotations of MGP voices as primary sources by ensuring that the researcher’s interpretation harmonizes with the original use and intention of MGP voices. We even consider it as a duty for researchers to return to their research subjects, to assure that MGP are able to comment on the researchers’ selection of primary sources about their critical agency toward their experience of being marginalized in business. Hopefully this freedom of MGP to comment on the selection of primary sources can provide better access to MGP’s original criticism of their experience of being marginalized in contemporary business, compared with secondary sources.

In the abovementioned nine studies, the authors quote MGP’s voices and thereby express a part of a primary source, usually appearing in the form of interviews. We argue that the focal point in these interviews are not the questions posed by the researchers, but rather the information in the insider perspective articulated through quotations of MGP’s criticism of their marginalized role in business. However, this criticism is being made possible by open and explorative questions articulated by a researcher intending to listen to what the person interviewed autonomously and freely want to say. Thus, these nine studies could contribute to the development of a normative epistemology and subsequently a more positive policy plan. The scientific value concerns MGP’s voices and critical agency regarding personally experienced marginalization in business being used as a primary source.

Mahalingam et al.’s (2019) study provides an example. The authors narrate how the December 2015 floods in the capital city of a southern Indian state “eroded the livelihoods and everyday dignities of people from all castes and social classes. The floods killed 250 people and displaced over 1.8 million Tamilians” (p. 213). The burden of cleaning the city and its houses fell to a large, stigmatized community of Dalits, previously known as untouchables. The authors held long conversations and interviews with Dalits, understanding them as primary sources different from secondary sources such as newspaper articles, blogs, YouTube videos, and social media posts. Correspondingly, the authors approached quotes from the conversations and interviews as significant or core parts of primary sources, expressing the essence of the topics they wanted to explore. Their interpretation of this essence in the primary sources showed that the stigmatized individuals reacted in two ways when confronted with the humiliating and undignified task for which the local state forced 25,000 of them to take responsibility. One the one hand, the authors described this dirty work as traumatizing and humiliating, obviously expressing the MGP’s critical stance toward the challenges:

The biggest problem was dead rats, chickens, and other animals, which were in water for a number of days. It was horrible and nothing equips you to clean this. I did not eat for few weeks. Some workers fainted. I had to sleep outside my house fearing the smell. Painful to think about it. (Mahalingam et al., 2019, p. 223)

An indirect sign of the authors’ high evaluation of quotes of MGP’s critical voices regarding personal experiences of marginalization in business is the use of paraphrasing. When the authors paraphrase, they try as conscientiously and honestly as possible to express the essence or core topics from firsthand accounts of personally experienced marginalization. They describe the destructive experience of the marginalization. However, the authors do not understand this paraphrasing as granting direct access to the destructiveness experienced by MGP. The paraphrased interpretation appears immediately after the quote to be clear about this conscientious way of approaching its essence, repeating the reaction of fainting confronted with the destructiveness of the work:

Dalit janitors felt that they were not prepared in dealing with very difficult conditions of cleaning. Several janitors fainted while performing their work, being unable to overcome the nature of death and destruction they were witnessing. When janitors remembered the work they had done, they experienced pain in describing the smells, sounds, and breakdown of spaces they dealt with. (Mahalingam et al., 2019, p. 223)

However, the authors also let the MGP express themselves freely regarding how they coped with the challenges of trauma and humiliation. This shines through in a quotation expressing possible pride about their janitorial labor as a critical agency toward the marginalization and subordination that they experienced:

I was cleaning a six-floor building. In each room, there was waste. Waste had piled up to six feet high piles in each room in the building. Laptop, TV, fridge, chair, sewer, and gutter water, everything had got mixed up. Earlier, the building looked like a dilapidated one-hundred-year-old structure. After I cleaned it, it was restored as a new, clean, modern building. (Mahalingam et al., 2019, p. 230)

The paraphrased interpretation appears immediately after the quote this time, too:

By providing an account of how he contributed to refurbishing a building that had become dilapidated into a clean, modern space, the participant is articulating the rehabilitative potential of janitorial labor. Janitorial labor as rehabilitation reshapes the discourse about the contribution of Dalits in the aftermath of the Chennai floods. We feel that this is a process through which Dalits reject their subordination in the caste order and try to lay claims as equal citizens. (Mahalingam et al., 2019 p. 230)

The paraphrasing aligns with Sen’s perspective, interpreting the self-respect of the Dalit janitors as a sign of their questioning and rejection of their subordination, and thus as a sign of their freedom of speech about the reevaluation of their work in the original situation. Regarding the intersectionality of their powerlessness as “untouchables,” this resistance and critical agency toward their subordination discloses their personal power of self-respect and thus modifies the degree to which they are powerless.

Consequently, the nine studies in which the authors enabled MGP to speak freely about personally experienced marginalization and express critical agency are important discussion tools for normative epistemology. They emphasize that quotations of MGP voices are, and should be, treated as primary sources, not least for critical agency, and, thus, a principium for the scientific interpretation of their positive and negative roles in contemporary business. When the voices of MGP are allotted the value of primary sources, MGP are also given a greater freedom of speech in science. This could be part of the described new journal publishing policy, opening the possibility for positive changes, as Sen and Drèze (2002) described, and thus potentially questioning types and grades of powerlessness.

Using this perspective, we intend to open a new space in business ethics research to contribute to changing the current tradition of interpreting MGP’s role in business. The next theoretical possibility we explore for MGP’s voices as a basis for scientific knowledge production is a participative perspective. MGP (and others) could strengthen a more positive publishing plan by programmatically welcoming and developing studies with a participative perspective.

### The Participative Perspective

The participative perspective could be designed as part of qualitative studies of MGP’s role in contemporary business, underscoring the normative epistemological aspect of these studies. Consequently, the question would be what type of participative perspective and concept *ought* to be used in qualitative studies to interpret MGP’s explicit voices of criticism toward their experience of being marginalized in business. Based on the empirical evidence and the theoretical foundation that the current paper builds upon, we promulgate that it would then be of great importance to facilitate MGP’s free expression of such a participative perspective concerning, at minimum, three significant dimensions: (a) their life experiences (not only their criticism of personally experienced marginalization but also their practical involvement in, and distancing from, these problems through actions); (b) their critical attitude toward the social complexity they are “trapped by”; and (c) their narration of specific social phenomena they resist (Grimen, 2004).

First, social scientists should consider the voices and actions of MGP as valuable sources for a participative perspective. However, we delimit our study to voices to ensure a sufficient depth of analysis as per the scope of the article allowed by the word limit.

This implies the exploration of a social world, which Grimen (2004) defined as “lived experience” (p. 246). In our context, this lived experience relates, for example, to how the indigenous Maasai people of East Africa, according to the UN, were threatened with extinction and how their leader acted as an entrepreneur, leading his people out of the vulnerable situation and into successful cooperation with the multinational corporation (MNC). Their leader acted based on life experiences, essentially calling into question, doubting, and rejecting (Sen & Drèze, 2002, p. 274) their economic, social, and political powerlessness, a differentiation in line with intersectionality:

Our lives were self-sustaining without the outside world. Our diet consisted of meat, blood, milk, honey and herbs from the bush. You know one cannot farm in the Savannah because of the wild animals and the type of soil. When we got ill, we used the herbs. All that has changed. Our diet has changed. The illness people have today sometimes requires modern medicine. We have moved from the blended life with the wild animals and we now live on the peripheral areas of the Mara. We have to buy our vegetables from the open market. We need money for medical care. We need money for clothing. (Olabisi et al., 2019, p. 7)

The cognitive dimension of this lived experience of being marginal in a modern society, even without considering the challenges the UN considers as presenting risks of extinction, entails many phenomena: particular values (e.g., self-sustaining lives versus dependence on the outside world, money), ideals (e.g., a blended life with wild animals), what MGP know and believe (e.g., their diet has changed and introduced illnesses requiring modern medicine), what they hope and strive for (e.g., money for food, medical care, and clothing), and what kinds of concepts are available to structure their experiences (see Grimen, 2004). These cognitive phenomena served as a basis for how they acted when experiencing the problems and possibilities in the transition from being at risk of extinction and from a status as non-stakeholders to being powerful primary stakeholders in the MNC.

The leader’s entrepreneurial abilities were partly based on an alertness from observing the struggles of his mother, amplified through their shared experiences during his childhood and expressed in an old decision:

Seeing my mother’s struggles caused me to think more about the plight of Maasai women. I felt that if my mother owned her own cows she could sell them and help me further my education. So, from the very early age, I decided that I would one day make it possible for Maasai children from poor families to get an education. (Olabisi et al., 2019, p. 12)

The decision had long-term effects. The leader’s entrepreneurial initiatives contributed to a significant expansion of cooperation with the MNC. Over a decade, the initial group of 20 employed Maasai women expanded to 1600, and they produced sandals in larger and larger volume. Revenue from the production transformed their extensive powerlessness into a more powerful collective existence (Gold, 2016). A young Maasai woman expressed the value of the partnership as follows:

Before Pikolinos came, the women were totally dependent on the men, but now they are independent and can pay school fees, buy food, etc. (Olabisi et al., 2019, p. 8)

The result of the cooperation was that the MGP navigated out of poverty into financial independence, educational progress, better livelihood, and political influence.

Olabisi and colleagues’ interpretation of the Maasai group aligns with critical race theory, underscoring the significance of entrepreneurship for the economic progress of minorities. However, Gold’s (2016) study shows that some MGP face larger barriers than others do, focusing on “the systematic record of racial disadvantage experienced by black Americans … that has restricted their entrepreneurial success” (p. 1714), a point of view that fits stories other than that of the success of this African MGP in the process.

To understand how MGP think and act critically toward the experience of being marginalized in business contexts, researchers should use the type of insider perspective we have illustrated (pp. 31–32) as point of departure/primary source for their interpretation. We understand an insider perspective as MGP’s explicit viewpoints of their critical agency (as the quotations of the Maasai leader show on pp. 31–32), to function as the primary sources for (our) scientific interpretation. We have earlier differentiated between three possible categories of primary sources of this type (pp. 25–26). As complex primary sources, their many different voices have the capacity to grant scientists important access to the wide array of lived experiences of MGP in contemporary business. As such, using their voices can also ensure MGP have access to and opportunity for freedom of speech, something scientific perspectives coming from the outside of such lived experiences cannot achieve.

When researchers make use of aforesaid primary sources, the former should return to their research subjects and allow them to comment on, modify if needed, and also change if necessary what type of primary source the researcher ought to take as point of departure and, thus, the information that the researcher aspires to portray about them (e.g., quotes). This would strengthen the opportunity for MGPs’ voices to be heard–and subsequently enhance the quality of the research.

Second, social scientists should consider their voices as valuable resources in the critical interpretation of complex social systems and their contradictions as they function in real life. This implies access to how human actions and opinions play out in different complex contexts. Concepts, models of ideas, values, and belief systems are interconnected in more or less coherent systems, with their inner logic, for example, linked to well-founded arguments for normative and religious assumptions. Social scientists must comprehend the logic of such systems of MGP to analyze their complexity, and they should use methods that allow such thought systems to appear (e.g., discourse analysis of their voice; Dunn & Eble, 2015), becoming visible through the voices of the social actors themselves. Of the 7500 articles that we reviewed, the following one of the nine studies provided good indications of the voices of MGP as access to two such complex systems occurring in real life.

Tlaiss (2015) argued in her study of Islamic female entrepreneurs that only by “the in-depth exploration of the complexity of Islamic business ethics, gender, and entrepreneurship, can Islam and its influence on globalization be better understood” (p. 874). As a presupposition for exploring this complexity, the author gave voice to Islamic female entrepreneurs on a range of questions regarding the role of Islam in their everyday lives and businesses. Quotations of one woman’s perspective were used as important, however indirect, access to information about the complex relationships between her original business practice and the critical function of its religious and ethical foundations, with her power as the horizon:

If Mohamad (p) allowed his wife (Khadija) to be a trader, then Islam is supportive of my business. I don’t think Islam per se is the problem. If we read the Qur’an properly, we can quickly understand that Islam is supportive of women. My problem is with the Muslim scholars who choose the conservative interpretation of things in a manner that suits them as men. When I interact with my male clients, this interaction is totally professional and work related and God knows what I am doing. Plus, when I meet male clients or suppliers, it is always in a public place. (p. 868)

This female entrepreneur seems to be powerless relative to participation in religious roles, and probably also concerning participation in leading business roles, which traditionally have been dominated by men in Muslim countries. However, when it comes to money, reasoning, education, and the informal authority of the courage to utter criticism, she seems rather powerful in her own context, according to our interpretation of this study. Meeks’ (2003) concept of high-status inconsistency might be a relevant approach. First, the female Muslim entrepreneur argued that the Prophet Mohammed allowed his wife to be a trader and, consequently, to cooperate with men as part of her business; thus, this allows the female entrepreneur to practice business in the same way. This well-founded critical reasoning is based on an interpretation of a complex system of norms and religious values (i.e., the Koran) and, as such, on the female entrepreneur’s use of a critical type of freedom of speech, as uttered in the quotation. For her, the Koran is a holy scripture with an inner logic to which she must be obedient, even if it that obedience involves sharp criticism of the male Muslim scholars who delegitimize the female entrepreneurial project. Second, this quotation, and others referred to by Tlaiss, also indicates that the Koran gives rise to complex interpretations (some female oriented) that support female Muslim business practices and are critical of other, more conservative, interpretations, typically represented by male scholars, that are critical of female interpretations of the Koran.

To grasp the complexity of belief, value, and norm systems, and the contradictions of these complexities, researchers should consider the voices of social groups in general, and of the MGP’s personally experienced marginalization as part of this complexity, as primary sources and, consequently, as expressions of their critical free speech in scientific studies regarding participating in this complexity. Tlaiss does so when she interprets this and a series of other analog quotations, affording important access to and expression of this complexity. Consequently, the critical voices of these women from a marginalized group offer access to and expression of their participation in the complexity of the social systems of Islam and business practice.

Third, researchers should consider the quotations as valuable in qualitative studies to describe *specific* characteristics of a social phenomenon. Comprehensive ethnographic studies from the last century featured this characteristic—for example, Malinowski’s study of the Trobriander society in the Pacific Ocean, Evans-Prichard’s study of the Nuer people in Sudan, Ardener’s study of the Bakweri ethnic group in Cameroon, Barth’s study of rituals among the Baktaman people of New Guinea, and Gluckmann’s study of the law system among the Barotse people in North Rhodesia (today Zambia). The aim of these studies was to describe a society, or part of a society, and the meaning constructions of the insiders in that society in as much detail as possible to demonstrate its emic (particular or unique) characteristics. Such an endeavor can also be seen in more contemporary ethnographic studies addressing the representation of MGP in modern work life (e.g., Mahadevan, 2015; Westwood, 1984).

Hamilton et al. (2019) argued that the everyday heroism of street cleaners in Northeast England was characterized by “a paternalistic notion of being prepared to care” (p. 898). Based on interviews with street cleaners, the authors underscored that this heroism could be expressed as “responses to specific calls for help” (p. 898), combatting the problems of everyday life:

We help everybody. It doesn’t matter who it is, you know, if they come to us and say “my wife has had an accident, she can’t pull the bin out, she’s broke her leg, would you please come in and get the bin?” We’d say “no problem.” We’d go in and get the bin, we’ll put it back and they’ll just come out and say “right” but then you get a message in the office [from them] saying “thank you very much for your help.” (p. 898)

As indicated earlier, when it comes to this group, the powerlessness is inconsistent. According to Hamilton et al. (2019), they seem rather marginal when it comes to education and political and public influence and, to some degree, concerning jobs and money, but not concerning the self-respect and spontaneous support of others and resistance to the everyday problems they face, as informal, moral authority.

Consequently, a qualitative perspective intended to grasp and give free voice to the particularity or uniqueness of social actors’ behavior ought to interpret such quotations in general, and MGP’s narration of personally experienced marginalization in particular, as primary sources, granting better access to this uniqueness than secondary sources can. Thus, such particularity represents another type of scientific value to which scientists have important access and that should be freely expressed through the voices of MGP. This is often not the case in many dominant scientific perspectives, because the researcher is often not equipped to provide access to insider perspectives as part of the produced or published text; for example, in quantitative survey research, the researcher has most likely not met or been in contact with the MGP studied. Furthermore, the researcher most likely does not know anything about the respondents’ (that is, MGP’s) lived experiences, thoughts, or opinions aside from the exact and non-context-based answers provided to a set of preconceived survey questions.

## Conclusion: Theoretical Contributions, Limitations, and Future Research

We conclude the paper by summarizing our theoretical contributions, highlighting some limitations, and proposing fruitful avenues for advancing theory and scientific knowledge production regarding MGP in theoretical business ethics research. We demonstrate that the focus on MGP remains limited in published articles in leading business ethics journals and that the marginalized are rarely heard from directly through the inclusion of their voices (i.e., speech acts)—which would offer readers access to their criticism, stories, narratives, lived experiences, social constructions, meaning attribution, and worldviews—as the basis from which to theorize about their role and criticism of modern business and organizations.

### Theoretical Contributions

First, we have discovered that although leading business ethics journals have published work regarding MGP, most did not use an insider perspective as a point of departure for scientific knowledge production. In fact, only nine out of approximately 7500 published articles feature their actual voices. Arguably, scientific knowledge production in extant research that is based on insider perspectives remains diminutive, with the consequence that MGP are largely excluded from said knowledge production. Second, none of the nine articles engaged MGP’s narration of personal experiences to theorize their marginalization in a business context.

In terms of the overall normative epistemology, we advance a case for producing scientific knowledge that is not based on the positivistic, linear research process that defaults to analysis of the lived experiences of research subjects primarily through extant theory and relies on deploying the many scientific instruments and preconceived concepts contained in the intellectual armory of the researcher. Our intellectual biases and lenses might result in analyses of the data shaped by what our theories make us see. For example, our theories’ limited ability to appreciate and capture “social reality” might lead to an inability to comprehend and convey the inherent complexities and context specificities of the lived experiences (Bourdieu, 2004; Grimen, 2004, p. 246).

Our article claims four additional contributions in terms of four areas of opportunities for theorization and scientific knowledge production regarding insider perspectives. In terms of marginalized theories, we highlight the relevance of less conventional avenues for theorizing, including a researcher’s own marginalized experiences and the marginalization that exists within academic institutions. (1) Furthermore, we recommend normatively facilitating MGP’s freedom of speech as a means of critical agency regarding their experiences, both as a pertinent source of theorizing and, in an iterative, inductive, and reinforcing fashion, as a goal to obtain increased access to their voices. (2) We also recommend a more positive gatekeeping role of Academia. We argue that *JBE* and *BEQ* ought to strive for a more positive publishing policy where these media platforms enable the critical agency of MGP. This would oppose MGP lacking traditional forms of power and support them as to produce resistance toward the lack of power they experience when being marginalized in business. (3) In terms of primary sources, we elucidate the possibilities for theorizing that would result from treating the voices of marginalized people themselves as the primary sources for the scientific interpretation of their personal experiences of marginalization in business organizations, based on their voices providing important access in the text. (4) Concerning a participative perspective, another opportunity for theorizing comes from access granted by the lived experiences of those being studied (that is, MGP) to the particularities and uniqueness of the social actors and their behavior in the marginalization of MGP in business, in addition to the corresponding complexity of their belief and behavioral systems.

The theoretical instruments associated with our contribution could enrich the understanding of ethics in business ethics research. Our literature review and normative epistemology have shown that business ethics studies ironically lack important perspectives on ethics because of their lack of a coherent epistemology in the study of MGP. This deficiency characterizes the most influential ethical theories, such as deontology, virtue ethics, discourse ethics, consequentialism, CSR, political CSR, and stakeholder theory. However, these theories could have been enriched in their understanding of ethics and its epistemological basis if their influential proponents, such as N. Bowie, R. C. Solomon, G. Palazzo, A. Scherer, and E. Freeman, had used the free speech of MGP and its critical agency to interpret MGP’s duties, virtues, discussions, actions and consequences, political responsibility, stakeholder dialogs, and engagement to inform a more profound exploration not only of the ethics of business corporations but also of the ethics of society.

### Limitations

First, we only analyzed articles published in the two leading business ethics journals: the *JBE* and *BEQ*. It is not unlikely that works on MGP—including some in which their voices are expressed (and potentially through other means)—have been published in other journals or outlets (e.g., books). However, as the two aforementioned journals are the leading outlets in the business ethics research field (according to journal ranking lists and the FT 50 journals list), we can plausibly expect them to exercise the most impact within the field and thus reflect the depth and breadth of the research in the field.

Second, the term and phenomenon of marginalization is contestable; thus, the sampling strategy of articles is also contestable. This means that some articles related to people who are marginalized and/or experiencing the phenomenon of marginalization might not have been included in our total universe of studies regarding MGP. Furthermore, the composition of our sampled articles might have been slightly different if we had not primarily focused on quotes/speech and/or the MGP’s lived experiences in our inquiry. In our theorizing, we subscribed to the definition of said people, presented in the Introduction, and focused on those being marginalized in relation to most variables.

Third, our concentration on quotations from MGP implies a concentration on speech acts; however, the viewpoints of MGP are also expressed through other means, such as artifacts, modern rituals, actions, and written documents, phenomena that could enrich the normative epistemology of MGP.

### Future Research

We propose fruitful avenues for future research in line with the normative perspective. First, given the limited number of studies on MGP, more research is needed to derive a deeper, more multifaceted understanding of the role of these groups in contemporary business. Second, we encourage more theorizing about MGP to understand the different degrees of impact on business from an included stakeholder perspective and from the perspective of ideal types in a Weberian sense, distinguishing between strong, medium, and small degrees of marginalization. Third, we strongly encourage examining what it means to be marginalized and the social construction of who constitutes these groups. This is important, as MGP should not only be treated as objects to investigate, as a fixed phenomenon; indeed, they are knowledgeable subjects capable of interpreting their worlds and the worlds of others and of processing others’ interpretations of their worlds, as our recommendation that MGP contribute to a new publishing policy in the *JBE* and *BEQ* clearly underscores. Thus, different forms of otherness and othering processes should be examined—from a cross-cultural perspective—as we cannot expect these constructs or phenomena to be equivalent across cultures, sociopolitical and economic contexts, or institutional practices (Guttormsen, 2018). The ethical dimensions of otherness in relation to MGP could be examined: “The essence is … that the Other is different and other from me and that I in my ethical acknowledgement of this otherness must let the Other disturb me” (Muhr, 2008, p. 180). This could be a disturbance of publishing policies.

Fourth, we see a need to contest extant theories from the perspectives of the marginalized, to explore whether the scientific understanding of key managerial and organizational behavior and thinking (e.g., sustainable innovation and business models, talent management, inclusion/exclusion, work–life balance) need to be nuanced because such research tends to focus on employee groups other than MGP. Fifth, we encourage conceptualizing MGP and their roles in business in relation to the emerging agendas of responsible research and innovation, as well as responsible management—in addition to the UN’s 2015 Sustainable Development Goals.

Sixth, on a methodological note, we encourage methodological innovation where the conventional lines between the researchers and MGP as research subjects are diminishing. This might take the form of research designs in which MGP are placed in the driver’s seat as fellow researchers and set the direction of what knowledge to produce about MGP phenomena and/or in collaborative ventures with scientists (e.g., collaborative ethnography). Finally, we see a need to investigate MGP and their roles in business in empirical contexts, such as during and after the ongoing pandemic, and the impact of context on their roles and marginalization, in addition to the rapidly changing nature of the future of work (Perkins et al., 2021)—preferably in a comparative perspective across organizations and countries.
